# One-Stage Combined Thoracic Ancient Schwannomas Total Removal and Coronary Artery Bypass

**DOI:** 10.1155/2011/497960

**Published:** 2011-09-25

**Authors:** Kadri Ceberut, Erdinc Naseri, Atac Celik, Ahmet Muslehiddinoglu, İsmail Ergin

**Affiliations:** ^1^Department of Thoracic Surgery, Tokat State Hospital, 60100 Tokat, Turkey; ^2^Department of Cardiovascular Surgery, Tokat Park Medical Hospital, 60100 Tokat, Turkey; ^3^Department of Cardiology, Gaziosmanpasa University, 60100 Tokat, Turkey; ^4^Gaziosmanpasa Universitesi, Arastirma Hastanesi Kardiyoloji AD, Eski rektorluk binası, 60100 Tokat, Turkey; ^5^Department of Pathology, Tokat State Hospital, 60100 Tokat, Turkey; ^6^Department of Radiology, Tokat State Hospital, 60100 Tokat, Turkey

## Abstract

Ancient schwannoma is a rare variant of neural tumors though rarely seen in the thorax. The combination with coronary artery diseases is also rare. Here we describe a 66 year-old male who had undergone one-stage combined surgery for thoracic ancient schwannomas removal and coronary artery disease. The masses were, respectively, 13 cm in the middle mediastinum and 5 cm in diameter originating from the intercostal nerve. The tumors were successfully removed using sternotomy, and then a coronary artery bypass grafting was performed. Here we discuss this rare tumor in relation to the relevant literature.

## 1. Introduction

Schwannomas are neurogenic tumors originating from Schwann cells of the neural sheath. Ancient schwannoma is a rare type of schwannoma that tends to grow slowly. The term “ancient” is proposed to describe neural tumors with long duration showing degenerative changes. Schwannomas are usually benign tumors, but the marked nuclear atypia of the tumor often leads to erroneous diagnosis of malignancy. Schwannomas frequently originate from the extremities, head and neck, and are most often localized in deep sites such as the retroperitoneum. They are rare in the thorax.

## 2. Case Presentation

**Figure 1:**
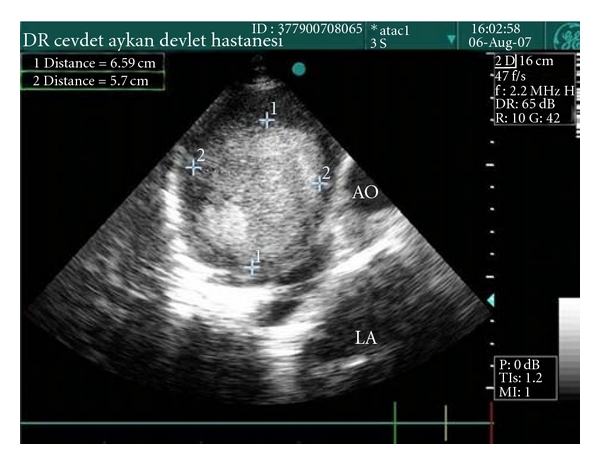
Paracardiac mass on transthoracic echocardiography.

**Figure 2:**
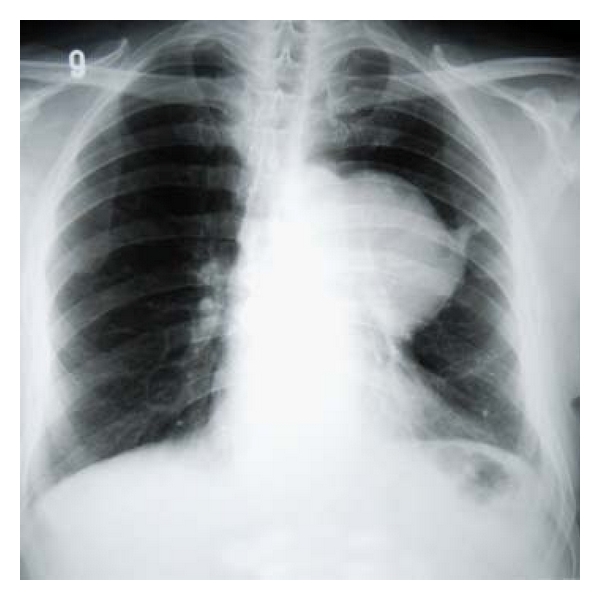
A well-circumscribed mass at the middle lung field.

**Figure 3:**
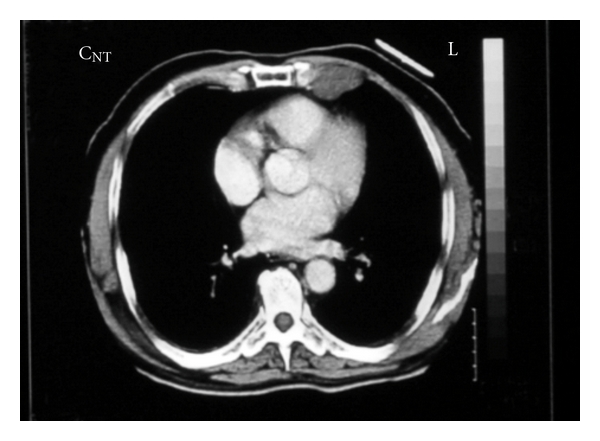
Computed tomography shows a homogeneous large cystic mass with a small solitary nodular area at the inferior margin.

**Figure 4:**
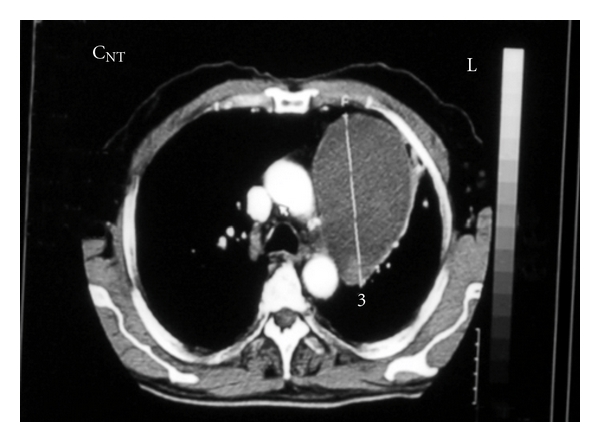
Computed tomography shows homogeneous cystic mass at the costosternal junction.

A 66 year-old male patient had been followed for nearly two years with a mediastinal mass. Two years ago a mass was observed near the left cardiac margin measuring 6.5 × 5.7 cm in a modified left parasternal view of the transthoracic using echocardiography (Figure 1). Further radiological evaluation had been offered but the patient had refused any further evaluation or surgical intervention. Nearly two years after the initial diagnosis, the patient was readmitted to thoracic surgery department for further evaluation. On chest roentgenogram, a well-circumscribed mass in the middle zone of the left lung field was observed (Figure 2). Chest tomography showed a well-circumscribed huge cystic mass measuring nearly 11 × 8 cm neighboring the aortic arch and left pulmonary artery, and a small cystic mass approximately measuring 5.0 cm on the sixth costosternal junction. A small nodular solid area was observed at the inferior margin of the huge cystic mass. These two masses were both well defined and homogeneous (Figures 3 and 4). During preparation for surgery, angina pectoris developed, and a cardiologic evaluation was performed. The patient underwent coronary angiography, and an atherosclerotic lesion causing significant stenosis was found in proximal and mid portions of left anterior descending artery and left circumflex artery. After the coronary angiography, a decision was made to undertake surgery in order to remove the tumors and to perform coronary artery bypass grafting.

**Figure 5:**
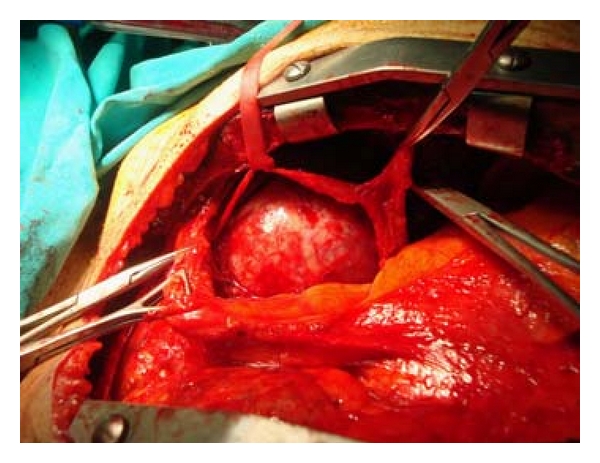
Operative view after sternotomy and preserving nervus vagus; huge mass occupying aortic arch.

In preoperative evaluation, we considered it possible that the mass dwelling on the aortic arch may make it impossible for proximal anastomoses of coronary bypass. In order to avoid this complication, we decided to first remove the mass, then perform coronary artery bypass grafting using sternotomy, in a one-stage combined operation. The chest was opened by sternotomy, and a huge encapsulated mass was observed dwelling the aortic arch and the left pulmonary artery with a proximal extension to the ascending aorta (Figure 5). After preserving the nervus vagus, the huge mass was removed by blunt and sharp dissection. The smaller tumor that originated from the intercostal nerve was also removed from the sixth costosternal junction. Following this phase, the ascending aorta became available for the proximal anastomoses of the saphenous vein grafts. Thereafter coronary artery bypass grafting was performed (sequential left internal thoracic artery proximal and mid left anterior descending artery, aorta saphenous circumflex.) Perioperative findings confirmed our preoperative suspicions. 

**Figure 6:**
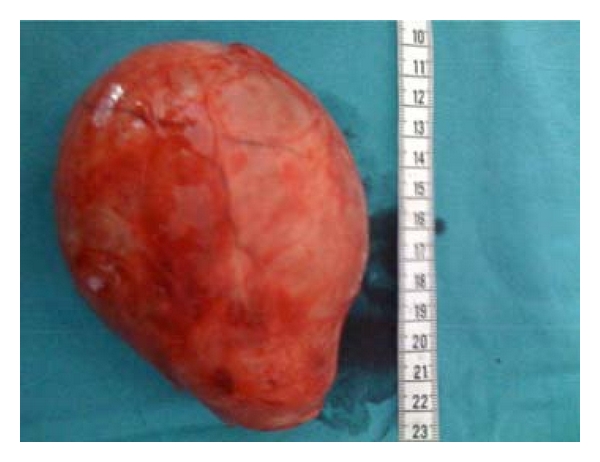
Resected encapsulated mass.

**Figure 7:**
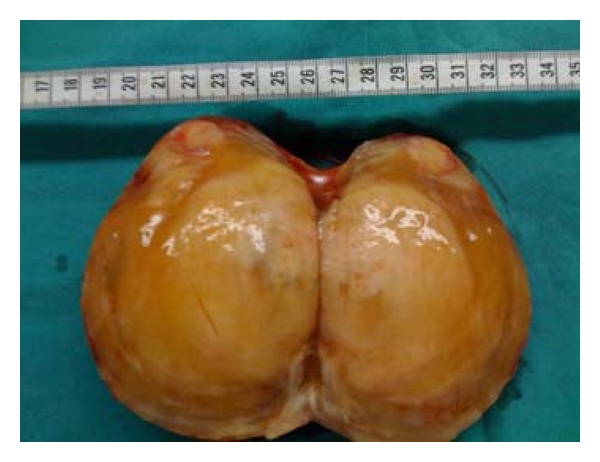
Cut surface of the mass; gelatinous, tan/yellow appearance with a solid area at the edge.

The gross appearances of these two masses were alike. They were reddish, bright, encapsulated masses measuring 13 cm and 5 cm in diameter, respectively. The cut surfaces had a bright, tan/yellow, gelatinous appearance, and a small solid area was observed in the larger one (Figures 6 and 7).

**Figure 8:**
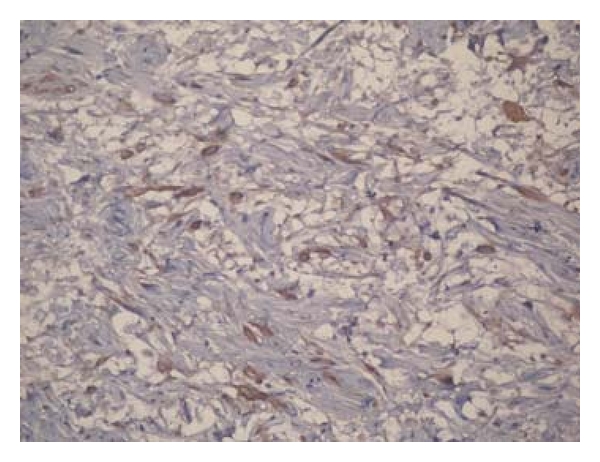
Atypical cells in hypercellular area (H&E; 50).

**Figure 9:**
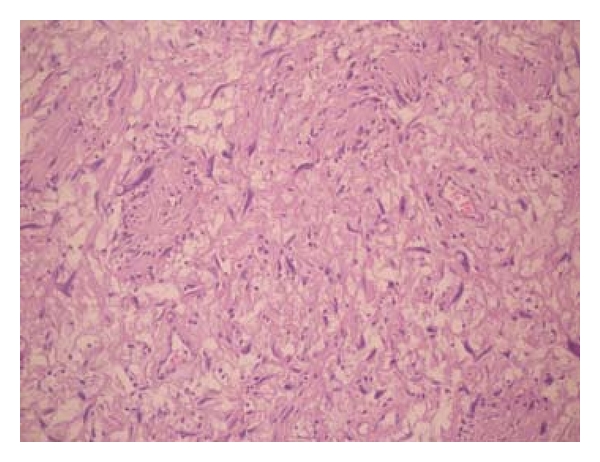
S-100 positivity in atypical cells.

On histological examination degenerative changes and atypical nuclear features were noted, but a mitotic figure was lacking. Immunohistochemical analysis was positive for S-100 protein (Figures 8 and 9).

The patient was discharged on the sixth day after the operation with a slightly elevated left diaphragm. Voice hoarseness was observed on control examination. On further control examinations, the elevation of the diaphragm had gone back to normal and the voice had improved. Whole body examination to exclude the possibility of Von Recklinghausen's neurofibromatosis revealed no additional tumors. The patient recovered fully one year after the operation; recurrence and metastasis were not observed.

## 3. Discussion

Ancient schwannomas are encapsulated tumors with long duration and a benign nature, showing degenerative changes. Malignant transformation is rare. Few cases of thoracic ancient schwannoma have been reported in the literature [1–5].

These tumors tend to develop slowly and seem to be symptomless until they reach a sufficient size to compress adjacent structures. They may be detected incidentally on routine roentgenograms. Our patient was asymptomatic until cardiac complaints began, and a paracardiac mass was detected by transthoracic echocardiography.

Schwannomas usually arise in the base of the spinal nerves but sometimes involve the thoracic nerves. They are often single lesions, but multiple tumors along a nerve may also occur. They may occasionally be associated with bone, skin, and central nervous system lesions (Von Recklinghausen's neurofibromatosis). Malign transformation risk is higher in patients with Von Recklinghausen's disease, but a solitary neurofibroma rarely tends to be malignant [4, 5]. 

The radiological features of ancient schwannomas have not been well described due to their rarity [6]. Long-term progression of the tumor leads to degenerative changes such as cystic formation, calcification, hemorrhage, and hyalinization. Ancient schwannomas have a characteristic appearance on computed tomography and magnetic resonance imaging, consisting of encapsulated solid components with cystic areas or appearing as cystic masses with marginal crescent-shaped or nodular solid components often containing calcifications [7]. In our case, computed tomography revealed cystic masses and a low-density area on the inferior margin probably due to hemorrhage as a degenerative change.

Some authors suggest that diagnosis of ancient schwannoma be considered when a patient presents with a hypervascular soft tissue mass containing amorphous calcification on plain graph and cystic areas on magnetic resonance imaging. They suggest that soft tissue calcification which is visible in a plain graph may be a characteristic feature of an ancient schwannoma [8]. As some authors have mentioned, calcification may not be characteristic of these tumors and in our case, we did not detect calcification neither on plain graphs nor on computed tomography [6, 7]. Magnetic resonance has been suggested to be much more useful than computed tomography in evaluating these degenerative features and defining the margin [6, 7].

Microscopically, schwannomas show a biphasic pattern with areas of highly ordered cellularity (Antoni type A) and with other less cellular areas where a copious myxoid matrix predominates (Antoni type B). Ancient schwannoma describes a tumor that has undergone degenerative changes with relative loss of Antoni type A areas and atypical nuclear features. The most significant histological features of these tumors are the presence of a high degree of nuclear atypia. Atypical cells show large, polymorphic, hyperchromatic, and often multilobed nuclei but lack mitotic figures.

Preoperative fine needle biopsy can be performed but an exact diagnosis may not be possible due to limited cellularity and the possibility of a mistaken diagnosis of malignant neoplasm due to degenerative histological changes [3]. Surgical excision of the mass is the gold standard of exact tissue diagnosis and treatment for these potentially resectable tumors. As we did, the chance of surgical excision must be assessed for this rare combination of these two diseases.
